# Liquid-Liquid Phase Separation in Nucleation Process of Biomineralization

**DOI:** 10.3389/fchem.2022.834503

**Published:** 2022-02-04

**Authors:** Da Qin, Zhen He, Peng Li, Shutian Zhang

**Affiliations:** Department of Gastroenterology, National Clinical Research Center for Digestive Disease, Beijing Friendship Hospital, Capital Medical University, Beijing, China

**Keywords:** liquid-liquid phase separation, biomineralization, nucleation, prenucleation clusters, polymer-induced liquid precursor

## Abstract

Biomineralization is a typical interdisciplinary subject attracting biologists, chemists, and geologists to figure out its potential mechanism. A mounting number of studies have revealed that the classical nucleation theory is not suitable for all nucleation process of biominerals, and phase-separated structures such as polymer-induced liquid precursors (PILPs) play essential roles in the non-classical nucleation processes. These structures are able to play diverse roles biologically or pathologically, and could also give inspiring clues to bionic applications. However, a lot of confusion and dispute occurred due to the intricacy and interdisciplinary nature of liquid precursors. Researchers in different fields may have different opinions because the terminology and current state of understanding is not common knowledge. As a result, our team reviewed the most recent articles focusing on the nucleation processes of various biominerals to clarify the state-of-the-art understanding of some essential concepts and guide the newcomers to enter this intricate but charming field.

## Highlights

• Biomineralization is an amazing phenomenon in nature. The inner structures of these biominerals are mostly beautiful and highly organized, which implied the complex crystallization processes, particularly in nucleation sections.
• Nucleation theories based on LLPS are becoming popular. PILP was the first theory proposed to identify the liquid-like nature of the biomineral precursors induced by acidic polymers.
• Some researchers found that LLPS could be an inherent property of calcium carbonate when nucleating, and polymers act as participants of the LLPS process.
• PNCs theory was raised to deeply explain the properties of precursors on smaller scales. However, the question about whether the PNCs aggregate through LLPS remains unclear.

## What is Biomineralization

Biomineralization refers to a charming process describing the formation of various biominerals participated by complex interactions of organic and inorganic molecules (Berendsen and Olsen, 2015; Kim et al., 2016; Grünewald et al., 2020; Li et al., 2020; Schoeppler et al., 2021; Wu et al., 2021). It is a typical interdisciplinary subject attracting biologists (Muñoz et al., 2019), chemists (Yuan et al., 2019), and geologists (Sivaguru et al., 2018; Sivaguru et al., 2020; Sivaguru et al., 2021) to figure out its potential mechanisms, and also provide inspiring clues to bionic applications (Cho et al., 2018).

Biominerals are commonly highly organized. Their attractive architectures were mostly explained due to the induction of organic molecules to inorganic environment. For instance, the molluscan nacre is a kind of typical fascinating biomineral. So Yeong Bahn etc. (Bahn et al., 2017) were interested in its beautiful appearance and found that a matrix protein, pif80, could form Ca^2+^-pif80 coacervates through LLPS to stabilize and further regulate the release of PILP-like amorphous calcium carbonate granules in intracellular vesicles, which may reveal the main biomineralization mechanism of nacre. Gallstone disease, which was considered a pathological biomineralization process, has made millions of patients suffer from pain and surgery every year. Luis E. Munoz etc. (Muñoz et al., 2019; O’Neil and Kaplan, 2019) found that neutrophil extracellular traps could act like the “glue” that sticks biliary calcium and cholesterol crystals, promoting gallstones assembly. When suppressing the innate immune system, the formation and growth of gallstones were also significantly suppressed. Moreover, Wenge Jiang etc. (Jiang et al., 2017) observed that chiral architectures of calcium carbonate can be easily controlled by adding chiral acidic amino acids (Asp and Glu). When adding L- and D-enantiomers separately to supersaturated calcium carbonate solutions, the chiral toroid that calcium carbonate formed could spiral in the counterclockwise or clockwise direction, respectively. This research emphasized the crucial roles of organic molecules in the crystal growth part of biomineralization (Jiang et al., 2018).

These highly organized, attractive architectures indicate the ability of organisms to control crystal nucleation and growth, which has not been fully understood. Among all the self-assembly processes, nucleation characterizes the very first step of crystallization and could heavily affect further crystal growth. How the first crystal appears in solution or colloid, although has been studied for decades, remains unclear. It seems hard to figure out the global crystallization or biomineralization process without a better understanding of the nucleation process.

## Classical Nucleation Theory Is Facing Challenges

Two nucleation theories including classical nucleation theory (CNT) and non-classical nucleation theory were commonly used to explain crystal nucleation (Erdemir et al., 2009). CNT is the simplest theory to illustrate the nucleation process. Gibbs (Gibbs, 1878) firstly proposed this theory in the 19th century to describe the condensation process of supersaturated vapor into a liquid phase. After many efforts by other founders, CNT has been widely applied now to explain nucleation kinetics from solutions (Guo and Severtson, 2003; Qiao et al., 2019; Xu et al., 2021). It contained explanations to two situations including homogeneous nucleation and heterogeneous nucleation. Homogeneous nucleation illustrates a process that the formation of nuclei in supersaturated solution is a result of the stochastic fluctuations of monomer association. According to CNT, nuclei are assumed to be spherical, and the global free energy ( ΔG ) of nuclei could be expressed as Eq. (1).

$$\text{Δ}G=\;\frac{4}{3}\pi r^{3}\Delta G_{\nu }+4\pi r^{2}\gamma_{p}$$ (1)

The bulk energy ( $\Delta G_{\nu }$ ) is the driving force of nucleation and could decrease the global free energy, which is determined by temperature (T), Boltzmann constant ( $k_{b}$ ), supersaturation degree (S), and molar volume (v). $\Delta G_{\nu }$ could be expressed as Eq. (2).

$$\Delta G_{\nu }=\;\frac{-k_{b}Tln\left(S\right)}{v}$$ (2)

**FIGURE 1:**
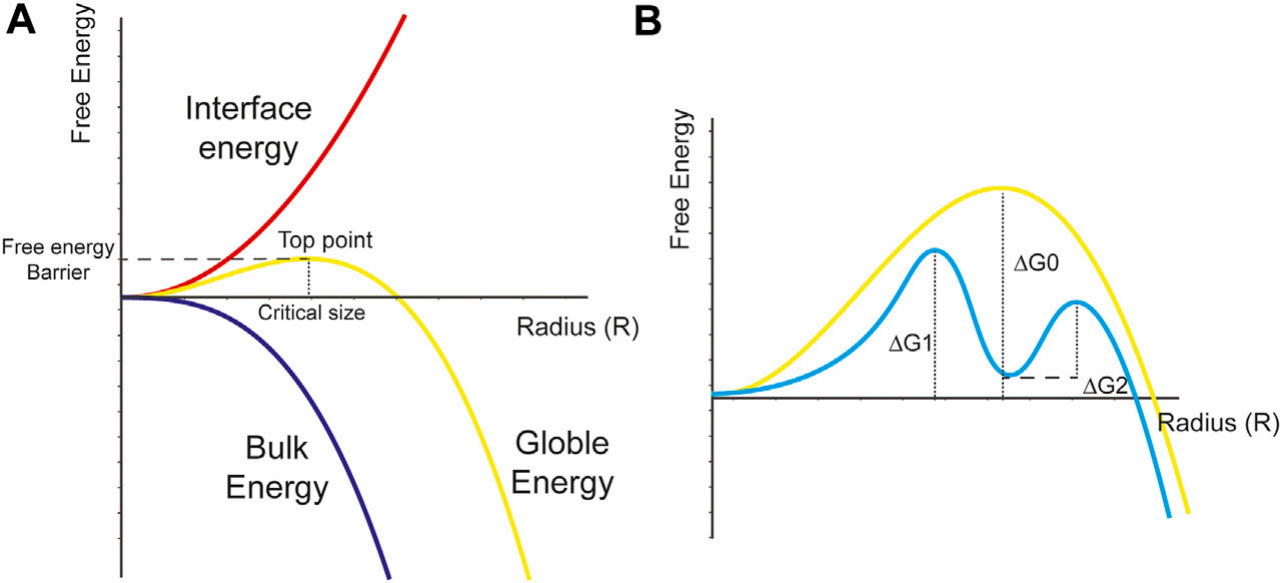
Diagrammatic sketch for classical nucleation theory.**(A)** The bulk energy is proportional to the cube of nuclei radius. Whereas the interface energy is proportional to the square of nuclei radius. The top point of global free energy is called the free-energy barrier, and the nuclei radius which could make the global system overcome the free-energy barrier is defined as the critical size. **(B)** The solute could firstly overcome the free energy barrier ( ΔG1 ) to achieve a metastable state. Solute in the dense droplets could then overcome the free energy barrier ( ΔG2 ) to accomplish the nucleation process.

The decreased extent caused by bulk energy is proportional to the cube of nuclei radius (r). The interface energy ( $\gamma_{p}$ ) is the resistance force of nucleation and could increase the global free energy. The increased extent caused by interface energy is proportional to the square of nuclei radius (Gibbs, 1878; Gebauer et al., 2014). The function curve (Figure 1A) is graphed according to this hypothesis. The top point of global free energy is called the free-energy barrier, and the nuclei radius which could make the global system overcome the free-energy barrier is defined as the critical size (Smeets et al., 2017). As is shown to us, to achieve the free-energy barrier, the ΔG should meet condition Eq. (3).

$$\frac{d\Delta G}{dr}=0$$ (3)

Taking Eq. 1 and Eq. (3) together into consideration, we can deduce that the critical size could be expressed as Eq. (4).

$$r_{crit}=\;-\frac{2\gamma_{p}}{\Delta G_{\nu }}$$ (4)

Then, the free-energy barrier that nuclei should overcome could be expressed as Eq. (5).

$$\Delta G_{crit}=\;\frac{16\pi \gamma_{p}^{3}}{3\Delta G_{\nu }^{2}}$$ (5)

As we know, most solid phase nucleation in biomineralization is heterogeneous. Heterogeneous nucleation illustrates the accelerated nucleation process due to the presence of foreign molecules which could act as heterogeneous nuclei and reduce the free energy barrier. The global free energy of heterogeneous nucleation could be expressed as Eq. (6).

$$\text{Δ}G=\;\text{Δ}G_{homo}f\left(m,x\right)\;\;\;\left(0\;\leq \;f\left(m,x\right)\leq 1\right)$$ (6)

In this equation, f(m,x)  represents interfacial correlation factor, and stands for the decreased degree of the free energy barrier caused by the appearance of foreign molecules. It is determined by interfacial interaction parameter (m) and the relative size of the foreign molecule, which ranges from 0 to 1. When f(m,x)→1 , it represents the poor correlation between foreign molecules and solutions, and the global free energy almost equals to that in homogeneous nucleation. However, when f(m,x)→0 , the free energy barrier is almost canceled, reflecting the promoted nucleation process compared to homogeneous nucleation.

Nowadays, along with the rapid development of the experimental instrument, a mounting number of researchers found the nucleation process that CNT predicted is not consistent with experimental results in numerous matters, no matter in homogeneous or heterogeneous nucleation, which may result from the oversimpliﬁcation of the nucleation model. Deniz Erdemir etc. (Erdemir et al., 2009) summarized some shortcomings of CNT, including the assumption that nuclei have uniform interior densities which is against numerous complex situations, the ignorance of the curvature dependence of the surface tension, the ignorance of collisions between more than two particles as well as two pre-existing clusters and so on. These challenges largely limit understanding of nucleation, and new theories need to be proposed to explain complex nucleation processes.

## When Non-classical Nucleation Theory Encounters LLPS

Non-classical nucleation theory demonstrates a process that before the formation of the crystal nuclei from the liquid phase, a metastable precursor phase firstly appears, and the crystal nuclei could next appear in this metastable precursor phase (Ma et al., 2017; Schreiber et al., 2017; Liu et al., 2019; Li C et al., 2021; Xu et al., 2021). Precursor phases could be shown in several forms including amorphous nanoparticles, droplets, complexes and so on.

**FIGURE 2:**
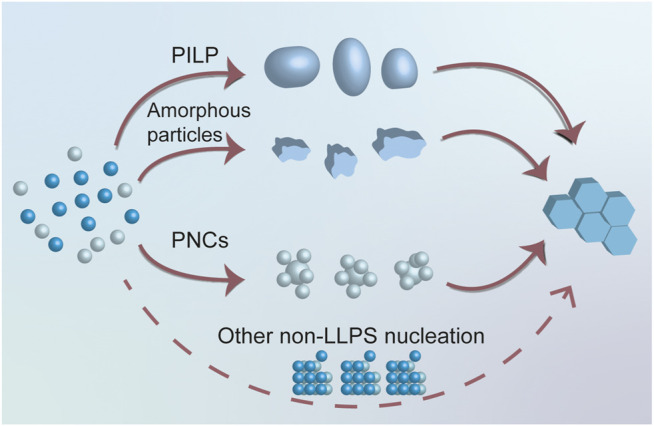
Diagrammatic sketch for nucleation processes. Among all nucleation processes, three LLPS dependent nucleation processes were identified, including PILP, amorphous particles, and PNCs.

Liquid-liquid phase separation (LLPS) is a popular interdisciplinary concept and is at the very core of the chemical (Freedman, 2020; Martin et al., 2020; Park et al., 2020; Riback et al., 2020; Iwata et al., 2021), biological (Ahn et al., 2021; Li RH et al., 2021; Deepankumar et al., 2021; Huang et al., 2021), and physical (Martin et al., 2021) processes of nature. Hundreds of high-level articles from many research fields focused on LLPS and tried to figure out its formation, performance, and regulation (Zhang et al., 2018; Alberti et al., 2019; Case et al., 2019; Gibson et al., 2019; Martin et al., 2020). In short, LLPS refers to a physicochemical process by which well-mixed fluid could separate into a dense phase and a dilute phase. With the rapid development of experimental tools, researchers found that many of solution precursors exist in the form of LLPS. This fact means that solute-rich droplets would firstly get separated from the whole solution, and could largely decrease the nucleation free-energy barriers. From a thermodynamic point of view (Figure 1B), the non-classical nucleation theory hypothesized that the solute could firstly overcome the free energy barrier ( ΔG1 ), which is much lower than the free energy barrier in CNT theory ( ΔG0 ), to achieve a metastable state. This state could be in the form of LLPS dense droplets, which has been observed by many researchers. Solute in the dense droplets could then overcome the free energy barrier ( ΔG2 ) to accomplish the nucleation process. However, a lot of confusion and dispute occurred due to the intricacy and interdisciplinary nature of liquid precursors. Researchers in different fields may have different opinions because the terminology and current state of understanding is not common knowledge. As a result, our team reviewed the most recent articles focusing on the nucleation processes of various biominerals to clarify the state-of-the-art understanding of some essential concepts and guide the newcomers to enter this intricate but charming field (Figure 2).

### Calcium Carbonate

**FIGURE 3:**
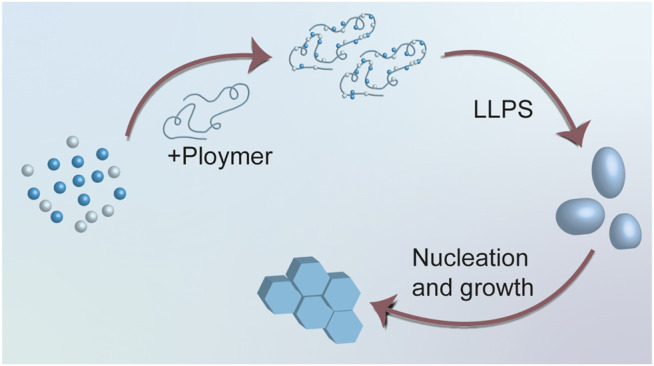
Diagrammatic sketch for PILP process. PILP theory hypothesized that polymer could sequester and concentrate the ionic species, and then form a metastable solution.

Calcium carbonate is the most studied component of biominerals. The study of its liquid precursor began in 2000. Laurie B. Gower etc. (Gower and Odom, 2000) firstly proposed the concept of polymer-induced liquid-precursor and began the period of studying liquid precursors. They added acidic polypeptides such as poly-aspartate into super-saturated calcium carbonate solution to simulate the induction functions of biomolecules, and *in-situ* observations showed that large droplets with distinct boundaries were subsequently formed. This phenomenon is then named PILP. Further investigations showed that different type of polymers resulted in different crystallization morphology, for example, addiction of poly-αL-aspartate commonly lead to streaks of a mosaic film of calcite, and poly-α, β, D, L-aspartate tends to produce thicker, isolated tablets. They explained the different morphology of crystals may result from various shapes of their liquid precursors. PILP theory emphasized the importance of the polymer, they hypothesized polymer could sequester and concentrate the ionic species, and then form a metastable solution (Figure 3). The liquid nature of the induced precursors was later identified by Wolf et al. (2017) using *in-situ* atomic force microscopy (AFM) in 2017.

**FIGURE 4:**
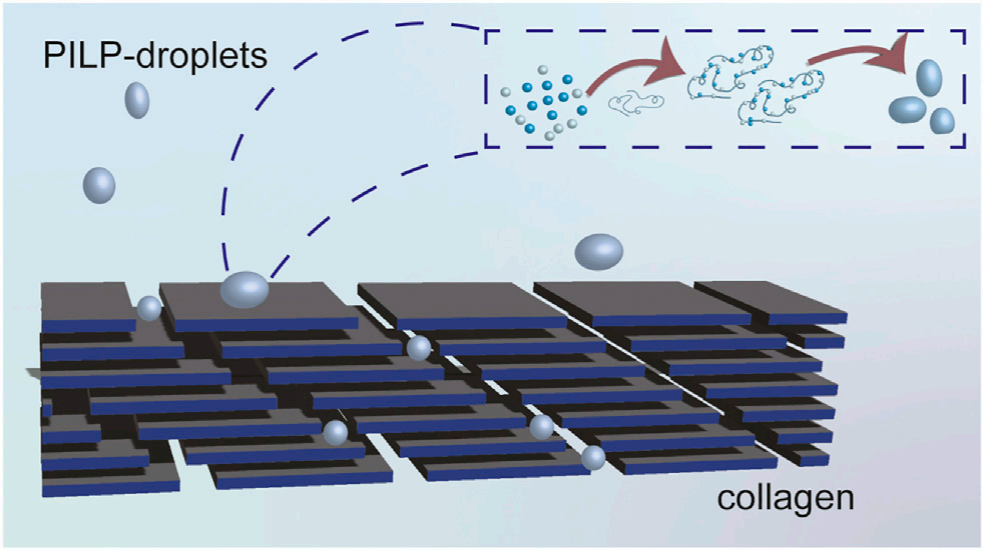
Diagrammatic sketch for collagen mineralizing. With the addition of polyacrylic acid, a liquid-like calcium carbonate precursor was first formed. Because of clear phase boundaries between PILP and solution, capillary force is easily generated. Then the liquid-like precursors could seep into collagen cracks and therefore result in space-filling crystals.

The concept of PILP raised the passion of researchers. Further research conducted by Olszta et al. (2003) demonstrated proof-of-concept of PILP theory by mineralizing type I collagen. Scanning electron microscopic (SEM) analysis was performed and illustrated the biomineralization processes as follows. With the addition of polyacrylic acid, a liquid-like calcium carbonate precursor was first formed. Because of clear phase boundaries between PILP and solution, capillary force is easily generated. Then the liquid-like precursors could seep into collagen cracks and therefore result in space-filling crystals (Figure 4). A similar phenomenon was also found in the sea urchin spine (Cheng and Gower, 2006).

However, the PILP theory wasn’t fully accepted by researchers. Some researchers (Bolze et al., 2002; Pontoni et al., 2003) began to explore to what extent the polymers are able to control the nucleation process. In 2004, Faatz et al. (2004) first proved that amorphous calcium carbonate (ACC) could get isolated from the whole solution via liquid-liquid phase separation process through a reaction of calcium chloride and carbon dioxide (which could produce calcium carbonate homogenously) observed by light scattering experiments and SEM analyses when the whole reacting system achieved proper concentration and temperature even without the participant of polymers. Wolf et al. (2008) provided more convincing evidence by designing an ultrasonic trap and observing with synchrotron X-ray scattering techniques in order to achieve a real-time and less-disturbance observation of the nucleation process. Their experiments further proved that ACC could increase in LLPS manners with the increasing super-saturation degrees without any additions.

These facts indicated that LLPS could be an inherent property of calcium carbonate when nucleating from a mother phase, and polymers could act as promoters or participants of the LLPS process, which could be particularly called PILP. Stephan E. Wolf etc. then introspected the potential connection between PILP and ACP, and took the eggshell of Gallus gallus as an attractive example to redefine the concept of PILP (Wolf et al., 2011). Firstly, their small-angle neutron-scattering (SANS) data showed ovalbumin could accumulate calcium ions to decrease the calcium activity of the bulk solution and increase the calcium concentration next to the protein, which could relieve the super-saturation status and thus stabilize the ACC phase. Then, electrospray ionization mass spectrometry (ESI-MS) was performed to prove that the ACC droplets were negatively charged and electrostatically stabilized. When adding negatively charged acidic protein such as ovalbumin (pI = 4.7) in this case, the LLPS phase could be stabilized and was similar to the PILP process that Gower etc. observed. However, when adding positively charged protein such as lysozyme (pI = 9.3), the whole system was destabilized and resulted in a strong coalescence. They assumed that the inner mechanisms of the PILP coating effect were the consequence of the destabilization of the ACC phase. This theory unified ACC and PILP theory and gave strong evidence to the regulation of organic polymers to inorganic matters. Furthermore, Gautron et al. (2021) summarized the roles of some specific proteins such as ovotransferrin (OVOT) (Panheleux et al., 1999), ovalbumin (OVA) (Dunn et al., 2012), ovocleidin-17 (Reyes-Grajeda et al., 2002), osteopontin (OPN) (Hincke et al., 2012) and the calcium-binding proteins (CaBPs) (Stapane et al., 2019; Stapane et al., 2020) which could perform an important function during avian eggshell biomineralization. These researches may solve the questions that how the chicken eggshell matrix proteins affect and regulate ACC mineralization and provided strong evidences to the hypothesis that organic polymers could regulate the biomineralization of inorganic matters.

**FIGURE 5:**
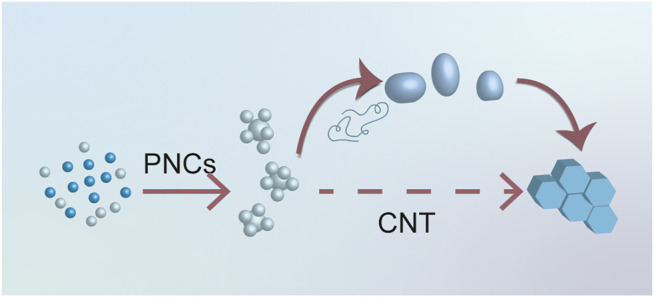
Diagrammatic sketch for PNCs theory. PNCs theory hypothesized that stable clusters aggregate and result in nucleation. Some researchers believed that the PNCs particles could aggregate and form LLPS-dependent structures, however, other researchers used the classical theory to explain PNCs’ aggregation.

The concepts of ACC and PILP based on LLPS theory have made some headway in exploring non-classical nucleation processes. However, these theories were mainly based on the observations and concentrate detections of the liquid precursors. To deeply understand the properties of these precursors, the hypothesis must be proposed on smaller scales. Pre-nucleation clusters (PNCs) theory was then raised by Gebauer et al. (2008) in 2008. A calcium ion selective electrode was used to measure the free calcium ions. They found that no matter at the undersaturated or the supersaturated stage, detected free calcium ions were always less than and in proportion to added calcium ions or carbonate ions. Considering the linear relationship between bound calcium ions and carbonate ions, they hypothesized that the calcium multi-binding during the prenuclear process happened, which was then called PNCs. After nucleation, further added calcium would be consumed by the growth of particles, and then forms a constant solubility concentration thermodynamically. Analytical ultracentrifugation (AUC) was further applied to indirectly prove their theories. Results showed that before nucleation, small size clusters could be identified with roughly 70 calcium and carbonate ions combined in a single cluster. Larger cluster species (hydrodynamic diameter ∼4–∼6 nm) could also be detected due to cluster aggregation. However, when it comes to the post-nucleation phase, the smaller clusters couldn’t be detected anymore. These facts supported the hypothesis that stable clusters aggregate and result in nucleation, which is quite different from the unstable clusters predicted by CNT (Figure 5).

How to illustrate the behavior of PNCs before nucleation, and how to explain the liquid-like property of PNCs aggregation bothered scientists for almost 10 years. If LLPS existed in the aggregation of PNCs remains unclear. Gebauer et al. (2008) only provided indirect evidence to the existence of PNCs and didn’t prove the relationship between PNCs and LLPS. The debate about whether the PNCs aggregate through LLPS still continues. Quite a number of researchers supported the opinions that PNCs aggregate through LLPS. In 2009, Pouget et al. (2009) employed cryogenic transmission electron microscopy (cryo-TEM) to prove the existence of oligomeric clusters in calcium carbonate solutions. Adam F. Wallace et al. (2013) used molecular dynamics simulations and observed a dense liquid phase within the concentration range in which the PNCs could be observed. In 2020, Avaro et al. (2020) proved that the solute PNCs could form phase-separated nanodroplets when crossing the binodal limit using potentiometric titrations and stopped-flow ATR-FTIR spectroscopy.

Multiple evidence (Habraken et al., 2013; Liu et al., 2015; Schreiber et al., 2017) seems to support the LLPS view of PNCs. However, many researchers disagreed with these opinions. For example, Henzler et al., 2018) used classical nucleation theory to predict the nucleation process of calcium carbonate PNCs and found out the great agreement with experimental data, which is against the LLPS view of non-classical nucleation way. So, identification of the property of PNCs still needs further direct evidence (Figure 5).

### Calcium Phosphate

Calcium phosphate (CP) is an important former of biominerals. CP could crystallize into hydroxyapatite (HAP) nanocrystals to become the main component of biominerals such as bones and teeth. However, the crystallization processes are still unclear. Research conducted by Olszta et al. (2003) demonstrated the complex interaction between calcium carbonate and type I collagen, giving proof-of-concept evidence for PILP theory. However, bones are mainly composed of collagen fibrils and HAP. Simulation of interaction between calcium carbonate and collagen couldn’t truly reflect the biomineralization process of bones. As a result, this team conducted deeper analyses (Jee et al., 2010). Acidic polypeptides (as simulations for non-collagenous proteins in bones) were first added into the mineralization CP solution, and then induced the liquid-like amorphous calcium phosphate (ACP) precursors. The synthetic collagen sponges were subsequently mineralized and their microscopic structures were comparable with bones. The higher molecule weight the acidic polypeptides were, the higher biomineralization degree and quicker reaction speed could be achieved. So, they concluded that PILP takes crucial parts of bone mineralization. This team also successfully re-mineralized the demineralized manatee bone *in vitro* to further prove their hypothesis (Thula et al., 2011).

Based on their research, a number of scientists focused on the biomineralization of bones and therefore deepened its understanding. In 2014, Rodriguez et al. (2014) realized that polymer simulation using poly-aspartic acid was far away from real biology condition. They made a lot of efforts to find out the director of bone biomineralization, and found osteopontin has the potential properties to be the inducer of PILP because osteopontin has a domain with consecutive aspartic acid residues, along with many phosphorylated residues, and the intrinsically disordered structures, which is easy to induce LLPS. Their experiments showed that osteopontin could direct intrafibrillar mineralization in a PILP-depend manner and could activate osteoclasts. Taking this study and the ACC study (Wolf et al., 2011) together into consideration, we can realize the roles that acidic polymers play in the PILP process are more likely to be directors or promoters than the inducers.

In 2020, He et al. (2020) reported a mechanistic study of the HAP mineralization pathways using *in-situ* transmission electron microscopy (TEM). They found that the mineralization of HAP is controlled not only by classical pathways but also by non-classical pathways. By classical ways, HAP was observed directly nucleated from the solution, while by non-classical ways, ACP could function as the substrate for HAP nucleation on the ACP surface. This study is meaningful and brought us new thinking about the relationship between CNT and non-classical nucleation theory, which might be dialectically unified rather than completely opposite, and could be coexisted in a similar situation.

The formation of some pathological biominerals is also associated with CP. Amos et al. (2009) reported that the formation of the Randall’s plaque may be related to the PILP process of ACP. They added poly-L-aspartic acid to the CP solution, and observed multi-laminated CP spherules formed through the PILP pathway by SEM. These spherules were striking resemble kidney stones, which give some clues that PILP could be a necessary process of kidney stone formation (Chidambaram et al., 2015; Lovett et al., 2019; O’Kell et al., 2019).

### Organic Biominerals

The nucleation process of organic matters is still mysterious to researchers. Many organic biominerals have not been studied. Among these organic biominerals, calcium oxalate seems to be one of the most common biominerals in nature. It represents more than 80% of the dry weight of some plants and is the main component of pathological biominerals such as kidney stones. Some researchers (Qiu et al., 2004; Hajir et al., 2014; Gehl et al., 2015; Kolbach-Mandel et al., 2015) identified that the amorphous phase existed before the nucleation of calcium oxalate. However, the nature of this pre-nucleation amorphous phase remains unclear. Ruiz-Agudo et al. (2017) tried to use PNCs theory to illustrate its property. Their results showed that calcium oxalate could form pre-nucleation clusters first in solution. The clusters grow by aggregation and could induce LLPS in this process, from which amorphous calcium oxalate nucleates. The whole nucleating process could be inhibited by citrate by colloidal stabilization effects. This study could help us understand the nucleating process of calcium oxalate, and may lead to the potential prevention strategy of kidney stones.

As is known to us, a large number of proteins could undergo LLPS depending on their intrinsically disordered regions (IDRs) and could condensate in a non-classical way, which may be responsible for a series of diseases. For example, the irreversible liquid-to-solid transition of microtubule-associated protein tau is tightly associated with Alzheimer’s disease (AD). Wegmann et al. (2018) observed the formation of tau droplets *in vivo* and *in vitro* and found that the intracellular LLPS of tau could lead to subcellular foci in a high concentration, which could be the structural basis of its physiological roles. Aberrant phosphorylation could lead to tau nucleation and thus causes AD. Zhang et al. (2020) observed protein tau phase transition in living cells. They found that the proline-rich domain of tau could drive and regulate LLPS through its phosphorylation, which may be the potential mechanism of AD. Similar to protein tau, protein FUS (Murakami et al., 2015; Monahan et al., 2017) and some RNA-binding proteins (Kato et al., 2012; Kroschwald et al., 2015) were also proved to undergo LLPS process and aggregate in a non-classical way.

Furthermore, a number of researchers began to study the roles of RNAs in LLPS. Because the structures of RNAs are sensitive to many factors such as salt concentration, pH, and ion composition, there is limited understanding on how RNA contributes to this condensate process (Roden and Gladfelter, 2021). Zhang et al. (2019) found that the G-quadruplex (GQ) forms of RNAs could trigger LLPS under physiological conditions, which provided the first evidence of the RNAs’ roles in triggering or maintaining LLPS. This may be the beginning of unveiling the mystery of RNAs nucleation.

The LLPS state is mostly unstable and is considered hard to achieve or maintain in nature, however, researches have shown that LLPS seems could be well utilized and regulated by cells, which is amazing and worth further studying.

There are still many problems unsolved about the nucleation process of organic biominerals, such as gallstones and atheromatous plaques. These structures are mostly composed of cholesterol crystals, which were poorly understood. Our team is focusing on the formation mechanism of gallstones and hopes to figure out the nucleation pathways of cholesterol.

## Bionic Applications

Biominerals are commonly highly organized. Their special structures could provide not only pretty appearance but also excellent mechanical performance as if done by the spirits of nature. To some extents, these biominerals have better properties than artificial materials. So, researchers studied bionic applications to mimic the biominerals (Wang et al., 2015; Picker et al., 2017; Huang et al., 2019; Yang et al., 2021). Laurie B. Gower etc. (Kim et al., 2007) first put forward the idea that PILP could be used for bionic applications in 2006. PILP theory indicated that polymers could induce liquid-like mineral precursors, and liquid precursors could be preferentially deposited onto the prepared functionalized templates. Connecting this property and the microcontact printing technique, their research provided the theoretical basis for further bionic utilizations.

Upon the basis of this feasibility test, a number of bionic applications were invented (Antebi et al., 2013). Li et al. (2012) used the PILP process to mineralize prepared densified collagen films, and produced stronger and more biocompatible biomaterials for bone grafts, which were comparable with bones. Their further research (Li et al., 2015) updated the mineralization template to thermo-responsive hydrogels composed of elastin-like recombinamers to achieve the controlled morphogenesis. The PILP precursors then crystallized to form close-packed crystals which were comparable to the bovine cortical bone because of the clustered porosity of the elastin-like temples. Other researchers (Wingender et al., 2016) also made an original contribution on mineralization templates and good effects were obtained. Moreover, Thula et al. (2010) compared the induction abilities of different polymers including poly-L-aspartic acid (PASP), poly-L-glutamic acid (PGLU), polyvinylphosphonic acid (PVPA), and polyacrylic acid (PAA). Then they found PASP and PGLU/PASP could yield nano-structured composites with the highest mineral content, which could be useful in bone grafts.

Besides the possible usage for bone grafts, the PILP process could also be used in the guided bone regeneration (GBR) and osteoporosis field. Wang et al. (2019) utilized homologous PILP processes and produced biomineralized membranes. The membranes are biocompatible with a high-stress strength, and could also promote the proliferation of bone cells. We are expecting their *in vivo* experiments, and hope that could bring new therapy strategies for GBR. Yao et al. (2019) also made exciting progress in the osteoporosis field. They designed a calcium phosphate polymer-induced liquid-precursor (CaP-PILP) material and injected the calcein-stained CaP-PILP droplets into the osteoporotic mice. They surprisingly found that the repaired bones of mice exhibited largely improved mechanical performance even comparable with the normal mice, which shed new light on hard tissue repair. Based on this research (Yao et al., 2019), Zhou et al. (2021) explored the effects of CaP-PILP injection on bone density and early implant osseointegration in mice. *In vivo* experiments showed that the CaP-PILP injection group had a superior bone repair and excellent implant osseointegration. This CaP-PILP material could possibly be applied as an adjuvant therapy to lower the bone-implant failure rate of osteoporotic patients.

Bionic applications of PILP in bones were not just limited to bone grafts. Because the PILP process could robustly produce intrafibrillar mineralization of collagen *in vitro*, which is quite similar to that formed in human beings, the minerals produced by PILP could be used as cell culture substrates for analyzing biological behaviors. For example, Choi et al. (2019) used this PILP-based bionic system and found that mineralization of collagen fibrils could reduce bone metastasis of breast cancer by resisting tumor cell adhesion, which provided potential prevention measurements for bone metastasis.

Bionic applications in dentistry were also popular and practical (Nurrohman et al., 2016; Nurrohman et al., 2017; Saeki et al., 2017; Saxena et al., 2018; Bacino et al., 2019). Similar to bionic applications of bones, Bacino et al. (2019) utilized PILP theory and found that the addition of poly-aspartic acid along with resin-modified glass ionomer (RMGI) could easily induce the PILP process and could be a feasible method to repair caries. Chen et al. (2020) tried to assess the repairability of CaP-PILP material (Yao et al., 2019) in caries dentin lesions. *In vitro* experiments showed that the CaP-PILP material could successfully induce remineralization of demineralized dentin collagen. A higher CaP-PILP could lead to an enhanced remineralization capacity.

The prevention of pathological biomineralization is a great part of these applications. A number of patients suffered from pathological biomineralization such as kidney stones and gallstones, and few useful prevention methods could be applied. Díaz-Soler et al. (2021) found that calcium oxalate precursors, which are the main component of kidney stones, could be stabilized by polyacrylic acid (PAA) by forming an LLPS-dependent PILP process. They found that PAA could stabilize the multi-ion complexes, and so that delaying the nucleation process. These facts may help to understand the pathological biomineralization process of kidney stones and give clues to its prevention.

## Concluding Remarks

Biomineralization is an amazing phenomenon in nature. The inner structures of these biominerals are mostly beautiful and highly organized, which implied the complex crystallization processes, particularly in nucleation sections. Among all nucleation hypothesizes, theories based on LLPS are becoming popular, and researches about the relationship between LLPS and the non-classical nucleation theory are increasing. So, we summarized them in the order of molecule types and timelines.

PILP theory was the first theory proposed to identify the liquid-like nature of the precursors induced by acidic polymers. Laurie B. Gower’s team discovered this phenomenon and lead related researches in theoretical and experimental levels for almost 20 years. Many biominerals were identified to undergo the PILP process in an LLPS-dependent way, and a number of bionic applications were also invented due to this theory. To clarify to what extent the polymers could affect the nucleation process, some researchers found that LLPS could be an inherent property of calcium carbonate when nucleating from a mother phase, and polymers could act as promoters or participants of the LLPS process, which could be particularly called PILP. This point of view sheds new light on the nucleation processes. PNCs theory was next raised to deeply explain the properties of precursors on smaller scales. However, the question about whether the PNCs aggregate through LLPS remains unclear, further direct evidence is still needed.

## Outstanding Questions

The interaction of organic molecules and inorganic molecules is complicated, through what regulation network the organisms could construct the intricate biominerals?

What genes control the nucleation-related organic molecules? Are there any transcriptional or post-transcriptional regulations inside this process?; How could we directly observe LLPS on the nanoscale?; Whether the PNCs aggregate through LLPS?

Organic crystallization is also common in human beings. Kidney stones are made of calcium oxalate crystals and gallstones are mainly composed of cholesterol crystals. These pathological biominerals are difficult to treat and are easy to recur. However, there is still no clear understanding in their nucleation and growth processes, especially the gallstones.

By understanding the nucleation process of gallstones, could it be possible to stop nucleating and prevent disease recurrence?

The applications of CaP-PILP in bones and teeth are successful, is it possible to finally apply CaP-PILP to treat patients?

## Abbreviations

AD, Alzheimer’s disease; ACC, Amorphous calcium carbonate; ACP, Amorphous calcium phosphate; AUC, analytical ultracentrifugationl; AFM, atomic force microscopy; CP, calcium phosphate; CaP-PILP, calcium phosphate polymer-induced liquid-precursor; CaBPs, calcium-binding proteins; CNT, classical nucleation theory; cryo-TEM, cryogenic transmission electron microscopy; ESI-MS, electrospray ionization mass spectrometry; GQ, g-quadruplex; GBR, guided bone regeneration; HAP, hydroxyapatite; IDRs, intrinsically disordered regions; LLPS, liquid-liquid phase separation; OPN, osteopontin; OVA, ovalbumin; OVOT, ovotransferrin; PAA, polyacrylic acid; PGLU, poly-L-glutamic acid; PILPs, polymer-induced liquid precursors; PVPA, polyvinylphosphonic acid; PNCs, pre-nucleation clusters; RMGI, resin-modified glass ionomer; SEM, scanning electron microscopic; SANS, small-angle neutron-scattering.
